# Understanding and removing surface states limiting charge transport in TiO_2_ nanowire arrays for enhanced optoelectronic device performance†
†Electronic supplementary information (ESI) available. See DOI: 10.1039/c5sc04076k


**DOI:** 10.1039/c5sc04076k

**Published:** 2015-12-08

**Authors:** Xia Sheng, Liping Chen, Tao Xu, Kai Zhu, Xinjian Feng

**Affiliations:** a College of Chemistry , Chemical Engineering and Materials Science , Soochow University , Suzhou 215123 , P. R. China . Email: xjfeng@suda.edu.cn; b National Renewable Energy Laboratory , 1617 Cole Boulevard, Golden , Colorado 80401 , USA; c Department of Chemistry and Biochemistry , Northern Illinois University , DeKalb , Illinois 60115 , USA

## Abstract

Charge transport within electrode materials plays a key role in determining the optoelectronic device performance. Aligned single-crystal TiO_2_ nanowire arrays offer an ideal electron transport path and are expected to have higher electron mobility. Unfortunately, their transport is found not to be superior to that in nanoparticle films. Here we show that the low electron transport in rutile TiO_2_ nanowires is mainly caused by surface traps in relatively deep energy levels, which cannot be removed by conventional approaches, such as oxygen annealing treatment. Moreover, we demonstrate an effective wet-chemistry approach to minimize these trap states, leading to over 20-fold enhancement in electron diffusion coefficient and 62% improvement in solar cell performance. On the basis of our results, the potential of TiO_2_ NWs can be developed and well-utilized, which is significantly important for their practical applications.

## Abstract 2

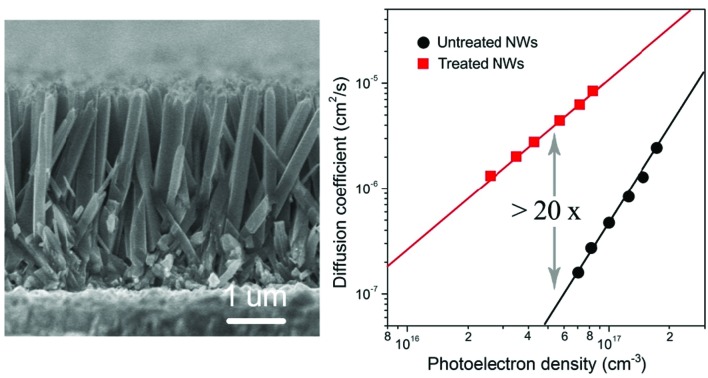
An effective wet-chemistry approach is demonstrated to minimize the trap states that limit electron transport in rutile TiO2 nanowire arrays, this leads to an over 20-fold enhancement in the electron diffusion coefficient.

## Introduction

Nanoscale semiconducting metal oxides have become promising low-cost electrode materials for solar cells, solar fuels and electric energy storage applications.^1^–^5^ Charge transport within electrode materials is a major determinant of device performance. It has been generally accepted that electrons undergo a random walk through electrode networks and are impeded mainly by surface trap states, grain boundaries and structural disorder.^6^–^9^ Compared to randomly packed rutile NP films, ordered single-crystal (grain boundaries free) TiO_2_ nanowire (NW) arrays are generally expected to have higher electron mobility, and have been the subject of extensive research.^10^–^14^ Unfortunately, measurements have shown that their electron mobility is not superior to that in NP films with the same phase (Fig. S1 ESI†).^15^ This implies that the influence of material architectures on electron transport is less evident in the presence of a large density of surface trap states.^8^,^9^ Thus, it is critical to understand the nature of the surface trap states and minimize them in order to exert the expected high electron mobility and device performance of NW arrays. In this study we reveal and demonstrate an effective way to remove the trap states that limit the electron transport in single-crystal rutile TiO_2_ NW arrays and their device performance.

## Results and discussion

**Fig. 1:**
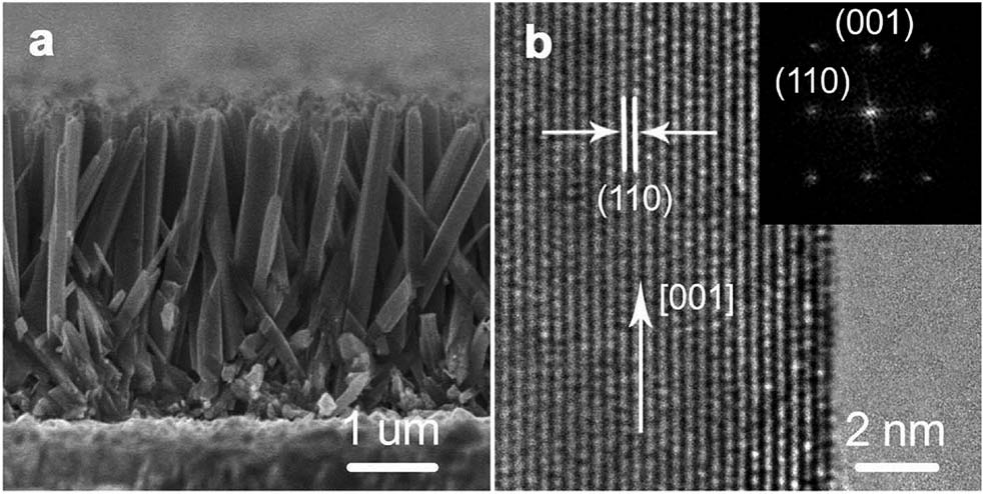
Morphologies of rutile TiO_2_ NW arrays. (a) Cross-sectional field emission scanning electron microscopy (FE-SEM) image of NW arrays with an average diameter and length of about 100 nm and 3 μm, respectively. (b) High-resolution transmission electron microscopy (HR-TEM) image of a typical NW. The NW has a growth direction of [001] and side surfaces of {110} crystal plane. The inset in panel (b) is the selected area electron diffraction pattern of the NW.

Aligned singe-crystal rutile TiO_2_ NW arrays were prepared *via* a conventional hydrothermal method.^10^–^14^ As shown in Fig. 1a, the as-prepared TiO_2_ NWs grow almost vertically from the substrate with an average diameter and length of about 100 nm and 3 μm, respectively. According to the high-resolution transmission electron microscopy (HR-TEM) image (Fig. 1b) and the selected area electron diffraction (SAED) pattern (the inset of Fig. 1b) analysis, the NW is highly crystallized and grows along the [001] direction with side surfaces of {110} crystal plane. Surface treatment is commonly used to boost the performance of semiconductors.^16^–^18^ In this report, the as-prepared TiO_2_ NW arrays were then processed with a wet-chemistry treatment by immersing in a H_2_O_2_–NH_3_ (aq) (10 : 1 v/v) solution at room temperature for different times, then rinsing with a copious amount of distilled water. Subsequently, both the treated and untreated NWs were annealed at 723 K for 30 min in an oxygen-rich environment.

**Fig. 2:**
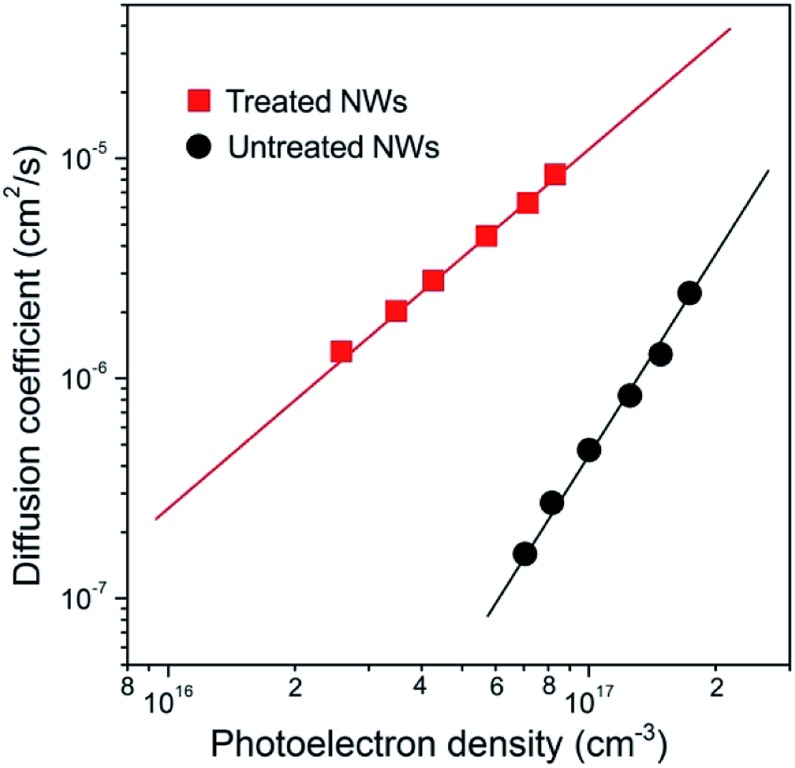
Electron transport properties of NW arrays. Dependence of electron diffusion coefficients (*D*) on the photoelectron density (*n*) for untreated (black line) and treated (red line) NW arrays. The electron transport in NW arrays is found to be over 20-fold improved after the wet-chemistry treatment.

Electron transport in these NWs was probed by using intensity modulated photocurrent spectroscopy (IMPS) and the results are shown in Fig. 2. The values of the electron diffusion coefficient (*D*) and photoelectron densities (*n*) are determined from the transport time constants (*τ*_c_) and film thickness (*d*) using procedures described elsewhere.^19^ Compared to the NWs without treatment, the *D* value in treated NWs was enhanced by over 20 times, for example, at a given *n* of 1 × 10^17^ cm^–3^. According to the previous study, the transient photocurrent response revealed from IMPS measurement is dominated by electron transport within electrode materials and can be explained by a trap-assisted diffusion model. The relation between *D* and the total trap density (*N*_T_) can be described using the following equation:^19^*D* = *C*_1_(*N*_T_)^–(1/*α*)+(1/3)^*n*^(1/*α*)–1^where *C*_1_ is a constant, and *α* is related to the shape of the distribution of the sub-bandgap trap states. Since 1/*α* is larger than 1, the existence of traps with a distribution of various energies is detrimental to the transport. Best fits to the data shows that *α* = 0.37 and 0.26 for NWs with and without treatment, respectively, suggesting that the shapes of the distribution of sub-bandgap trap states are different. A smaller *α* value indicates a longer tail and a relatively deeper distribution in trap energy levels, which is normally associated with the existence of deeper level traps that not only affect the magnitude but also the slope of the mobility–light intensity curve.^6^,^20^,^21^ Fig. S2† shows the dependence of *n* on voltage for the two cells based on NWs with and without surface treatment. The *n* value of untreated NWs is about 1.5-fold higher than that of treated ones at the same voltage, suggesting the total trap density is larger for untreated NW samples. Based on the analysis described above, one can conclude that (1) the slower electron transport in the untreated rutile TiO_2_ NWs is attributable to surface trap states with relatively deeper energy levels; (2) the treatment can passivate/remove the surface traps especially those with relatively deeper energy levels, leading to shallower distribution trapping energy (larger *α* value) and faster electron transport for surface-treated NW samples.

**Fig. 3:**
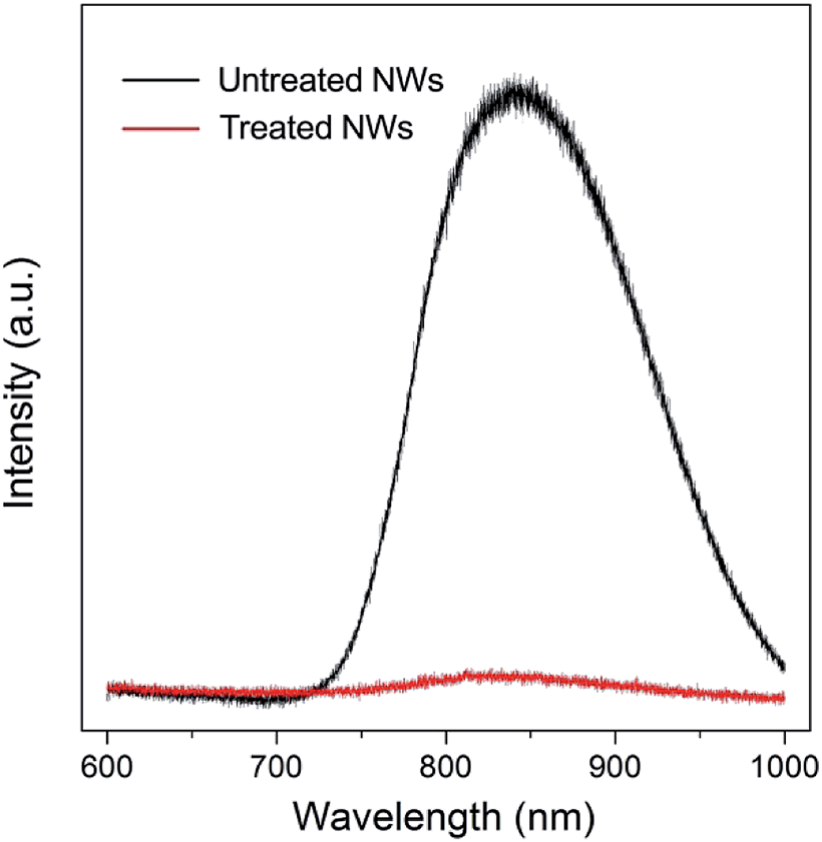
Photoluminescence (PL) spectra of TiO_2_ NW arrays without treatment (black line) and with treatment (red line). The untreated NWs exhibit a strong NIR PL peak centered at about 835 nm, while the PL peak intensity is significantly reduced for the NWs after treatment in H_2_O_2_–NH_3_ (aq) solution for 10 min.

Surface trap states are commonly associated with surface defects. N-type rutile TiO_2_ is usually in a nonstoichiometric reduced form that has intrinsic defects including oxygen vacancy and titanium interstitial (Ti_int_^3+^).^22^,^23^ Oxygen vacancies are thermodynamically unstable and can be readily eliminated *via* simple oxygen annealing at elevated temperature.^24^,^25^ Regarding the Ti_int_^3+^, it prefers to have a high coordination and is mainly located in the bulk. However, recent studies on rutile TiO_2_ (110) surface using atomic-resolution scanning tunneling microscopy have shown that Ti_int_^3+^ defects can diffuse from the bulk to the surface at temperature higher than 400 K and result in surface reconstruction.^25^–^28^ Photoluminescence (PL) is a highly sensitive technique for investigating surface characteristics of semiconductors. As shown in Fig. 3 (black line), a strong near-infrared (NIR) PL peak centered at around 835 nm was observed from untreated NWs. Such a PL peak (835 nm) of rutile TiO_2_ has been indexed to surface Ti interstitials, specifically to the Ti_int_^4+^.^29^–^31^ Since the rutile TiO_2_ NWs were prepared at a temperature of 453 K, and that their side surfaces are {110} crystal plane, Ti_int_^3+^ can diffuse to the {110} crystal plane. When the NWs were further annealed in an oxygen-rich environment, these outward-diffused Ti interstitials will be oxidized and form added islands that occupy preferentially at the surface interstitial sites, which results in the strong NIR PL spectrum. Such interstitial defects will likely lead to the formation of trap states in relatively deeper energy levels that extend virtually all the way to the conduction band edge. It is worth noting that, unlike oxygen vacancies, surface Ti interstitial defects cannot be easily removed *via* conventional oxygen annealing treatment; in contrast, oxygen annealing will promote their outward-diffusion and surface reconstruction process.

Fig. 3 (red line) shows the PL spectrum of TiO_2_ NWs after 10 min wet-chemistry treatment in H_2_O_2_–NH_3_ (aq) solution followed by 30 min oxygen annealing at 725 K. The peak intensity is significantly reduced, implying that the vast majority of surface Ti interstitial defects were removed after the treatment. Based on the differences of PL intensity and electron diffusion coefficient of NW arrays with and without treatment, it can be concluded that the surface Ti interstitial defects related trap states limit the electron transport of ordered single-crystal TiO_2_ NW arrays.

During the wet-chemistry treatment, H_2_O_2_ can react with surface Ti_int_^4+^ and form the soluble complex (TiO_2_·H_2_O_2_) *via* a possible chemical reaction:^32^ Ti_int_^4+^ + H_2_O_2_ + 2H_2_O → TiO_2_·H_2_O_2_ + 4H^+^. In the meanwhile, NH_3_ (aq) will neutralize the H^+^ and promote the forward reaction. The process of removing surface interstitials is rather fast as confirmed *via* PL studies. Fig. S3† shows the PL spectra of TiO_2_ NWs as a function of treatment time. The PL peak intensity was significantly lowered with only 30 s treatment. Upon the treatment, a Ti interstitial depletion layer near the surface will be formed, and consequently, their related trap states can be minimized. Crystal structures analysis including X-ray photoemission spectroscopy (XPS) (Fig. S4†), X-ray diffraction patterns (XRD) (Fig. S5†) and Raman spectra (Fig. S6†) of NW arrays with and without treatment remain unchanged, which confirms that no new phase or impurity was introduced during the treatment. Thus, the wet-chemistry treatment that is presented here is an effective approach to remove surface trap states in deeper energy levels, as exemplified by the observed over 20-fold enhancement in electron transport.

**Fig. 4:**
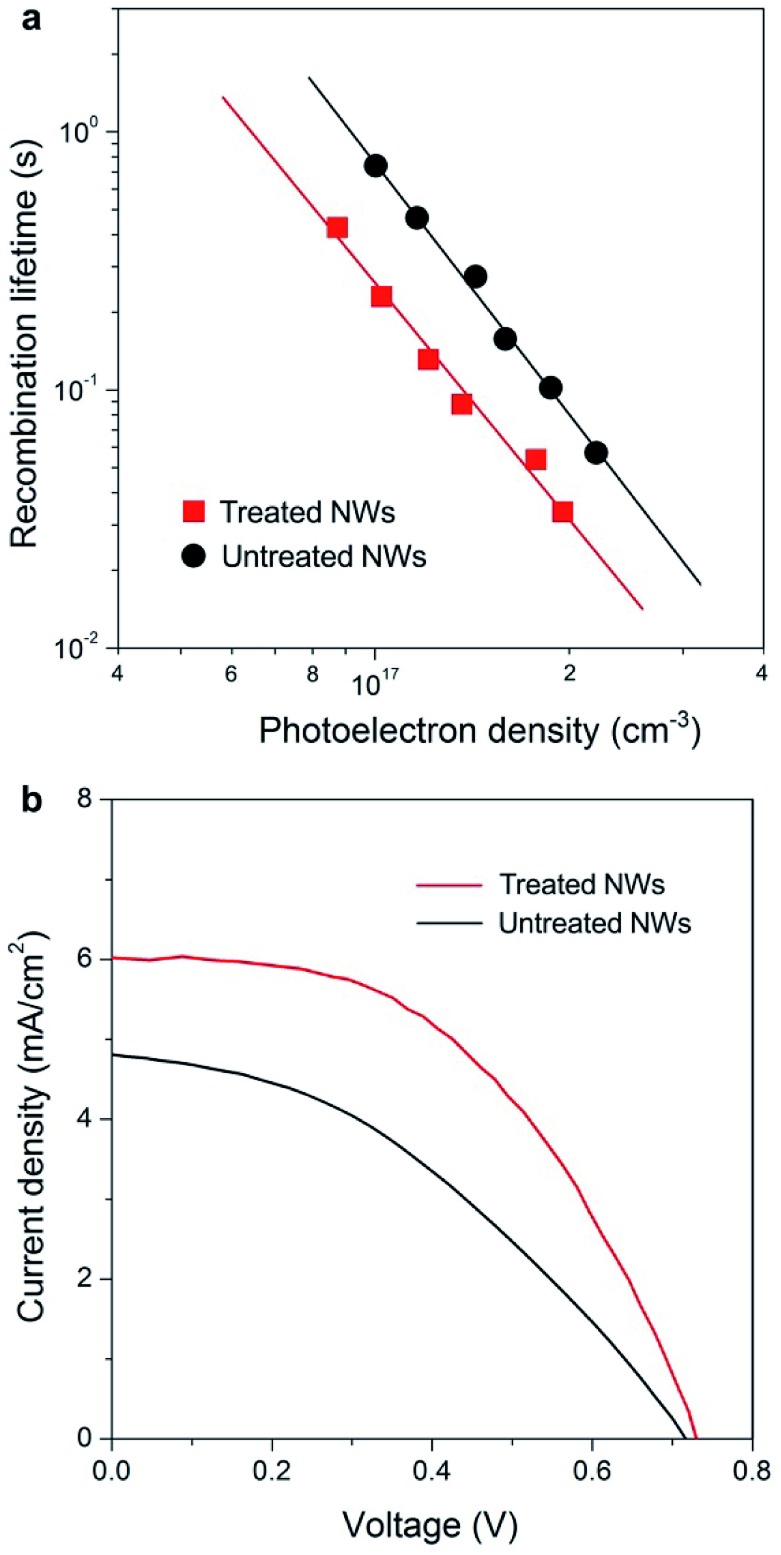
Performance of the NW array-based solar cells. (a) Comparison of the recombination times (*τ*_r_) as a function of photoelectron density *n* of solar cells based on untreated (black line) and treated (red line) NW arrays. (b) Current density–voltage characteristics of solar cells fabricated using untreated (black line) and treated (red line) NW arrays under simulated AM 1.5 light. The cell based on the treated NWs exhibits a short-circuit photocurrent density (*J*_sc_) of 6.01 mA cm^–2^, an open circuit voltage (*V*_oc_) of 0.73 V, and a fill factor (FF) of 0.50. In contrast, the cell based on untreated NW arrays shows a *J*_sc_ of 4.81 mA cm^–2^, a *V*_oc_ of 0.72 V, and a FF of 0.39. The photocurrent was measured using AM-1.5 simulated sunlight (Oriel Sol3A Class AAA Solar Simulator).

Fast electron transport is expected to lead to high electron collection efficiency and enhanced optoelectronic device performance. Fig. 4a compares the recombination times (*τ*_r_) as a function of photoelectron density *n* of solar cells based on the two samples. Base on the data in Fig. 2 and 4a, we determined that the electron diffusion length (*L*_n_) for treated NW is approximately 20 μm, 4 times longer than that for NWs without treatment (∼5 μm) using the relation of *L*_n_ = (*Dτ*_r_)^1/2^. The charge collection efficiency (*η*_cc_) of treated NW-based cells, as described by the relation: *η*_cc_ = 1/[1+(*d*/*L*_n_)^2^], where *d* is the electrode thickness, can be calculated to be 98%, which is 23% larger than that of untreated NW counterpart.

Fig. 4b compares the photocurrent density–voltage (*J*–*V*) characteristics of NW array-based solar cells under simulated AM 1.5 light. The cell based on NWs with faster electron transport rate exhibits a short-circuit photocurrent density (*J*_sc_) of 6.01 mA cm^–2^, 25% higher than that obtained on untreated NW-based cell (4.81 mA cm^–2^). In general, *J*_sc_ is determined by light-harvesting efficiency (*η*_lh_), charge-injection efficiency (*η*_inj_), and *η*_cc_. It worth noting that the surface areas of NW arrays with and without treatment are almost identical according to dye desorption results (Fig. S7†), suggesting that both *η*_lh_ and *η*_inj_ values of these devices are similar. Thus, the 25% improvement in *J*_sc_ of the treated NW-based cells should be mainly ascribed to the enhancement in *η*_cc_ of the electrode materials as described above. Besides, the treated NW-based cell exhibits a higher fill factor (FF), which can also be ascribed to the much enhanced electron transport property. Under similar surface areas, the faster electron transport leads to higher values of *J*_sc_ and FF, and 62% enhancement in solar to electricity conversion efficiency. In the future, if the growth technique could be extended to very long 1D ordered nanostructures then one would have a superior electrode material for various kinds of sensitized and heterojunction solar cells.

## Conclusions

In conclusion, we have revealed that the surface trap states of rutile TiO_2_ NWs in relatively deeper energy levels result in slower-than-expected electron transport. Moreover, we have demonstrated an effective wet-chemistry approach to remove these trap states, leading to over 20-fold enhancement in electron transport and 62% improvement in solar conversion efficiency. Considering the wide range of studies and applications of 1D single crystal TiO_2_ nanowire arrays, the significant charge transport enhancement achieved in this work will make it a real enabling technology for future solar cells, water splitting and electric energy storage device applications.

## Supplementary Material

Supplementary information
