# Supplementary material for: Understanding and removing surface states limiting charge transport in TiO2 nanowire arrays for enhanced optoelectronic device performance
Source: Chem Sci. 2015 Dec 8;7(3):1910–3. doi: 10.1039/c5sc04076k (PMC5966746; doi:10.1039/c5sc04076k)
Supplement: Supplementary file 1 [file SC-007-C5SC04076K-s001.pdf]

Electronic Supplementary Material (ESI) for Chemical Science.
This journal is © The Royal Society of Chemistry 2015

Supplementary Information

Understanding and Removing Surface States Limiting Charge Transport in TiO_2 Nanowires Arrays for Enhanced Optoelectronic Device Performance

Xia Sheng,[a] Liping Chen,[a] Tao Xu,[c] Kai Zhu, [b] and Xinjian Feng*[a]

a. College of Chemistry, Chemical Engineering and Materials Science, Soochow University, Suzhou 215123, P. R. China. E-mail: xjfeng@suda.edu.cn

b. National Renewable Energy Laboratory,1617 Cole Boulevard, Golden, Colorado 80401, USA.

c. Department of Chemistry and Biochemistry, Northern Illinois University, DeKalb, Illinois 60115, USA

Experimental Section

Aligned rutile TiO_2 NW arrays are synthesized via a conventionally hydrothermal method. In a typical experiment, fluorine-doped tin oxide coated glass substrates (Tech 8) are loaded into Teflon-lined stainless steel reactors (23 ml) that filled with 6 mL DI water, 6 mL 37% hydrochloric acid and 0.2 mL tetrabutyl titanate, and kept at 453 K for 3 h. The treatment of the NW arrays are processed as following: the as-grown TiO_2 NWs is immersed in a fresh H_2O_2 (30 wt%) /NH_4OH (25 wt%; v:v = 10:1) solution for 30 s to 10 min and then rinsed with a copious amount of distilled water. Both of the NW arrays samples with and without wet-chemistry treatment are finally annealed at 723 K for 30 min in oxygen-rich environment with the oxygen flow rate of 0.6 L/min before all measurements.

The structures of the samples are characterization using a field emission scanning electron microscopy (FE-SEM, S4800, Hitachi, Tokyo, Japan), high resolution transmission electron microscopy (HR-TEM, Tecnai F20, FEI, Hillsboro, OR, USA), and selected area electron diffraction (SAED). The crystal phase structures of the samples are investigated using an X-ray powder diffractometer (X'Pert PRO, PANalytical, Almelo, The Netherlands), X-ray photoemission spectroscopy (Escalab 250 Xi, Thermal fisher, USA) and Raman spectroscopy (Horiba, LABRAM HR, France). The photoluminescence (PL) measurement is used to investigate the surface characters recorded by a charge-coupled device (CCD, PIXIS: 100 BR,

Princeton Instruments, Trenton, USA) with a 325 nm line of He-Cd laser as the excitation light source. The untreated and treated NW samples for the PL measurement are grown on the high quality quartz substrates in order to avoid the interference caused from FTO substrate. Dye desorption characters is measured by UV-VIS spectra (Ocean Optics, Maya 2000 pro, USA).

For IMPS and IMVS measurement, the untreated and treated NW arrays are respectively coated with dye by immersion overnight at ambient temperature in a 0.5 mM ethanolic solution of commercial N719 dye. The electrolyte is composed of 0.8 M 1-hexyl-2,3-dimethylimidazolium iodide and 50 mM iodine in methoxypropionitrile. A conductive glass slide sputter-coated with 100 nm of Pt is used as the counter-electrode. The thickness of the electrolyte layer between the NW arrays and counter-electrode is fixed by the use of a 25 μm thick SX-1170 spacer (Solaronix). The photocurrent density and photovoltage of the DSSCs are measured with active sample areas of 0.24-0.5 cm^2 using AM-1.5 simulated sunlight produced by a 500 W Oriel Solar Simulator. Electron transport and recombination properties of solar cells are measured by intensity modulated photocurrent and photovoltage spectroscopies as described previously.[1]

1. K. Zhu, N. Kopidakis, N. R. Neale, J. van de Lagemaat, A. J. Frank, *J. Phys. Chem. B*, 2006, 110, 25174-25180.

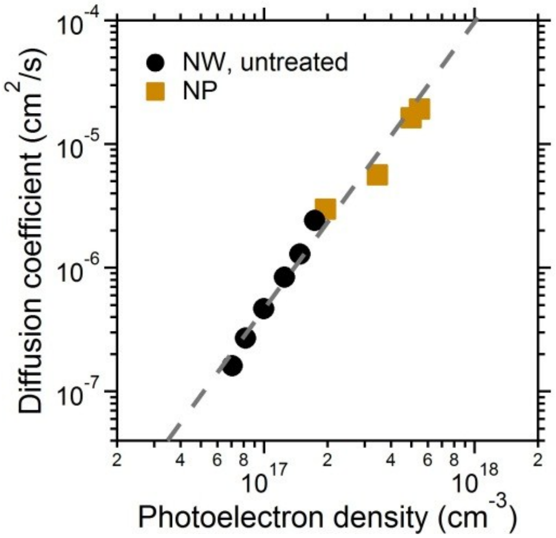

Fig. S1: The dependence of the electron diffusion coefficients (D) on the photoelectron density (n) for the as-synthesized NWs and NP films.

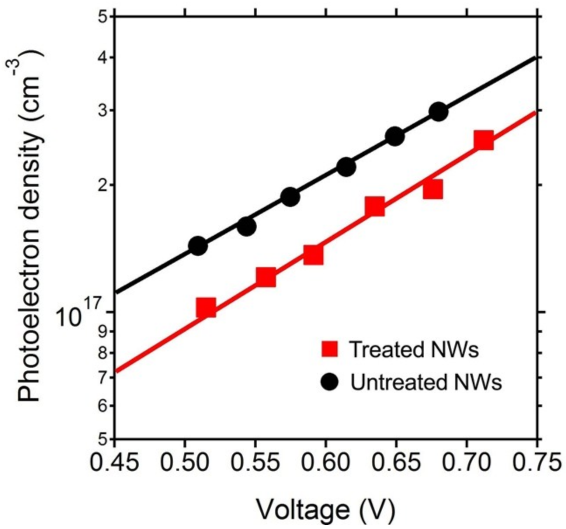

Fig. S2: The dependence of the photoelectron density (n) on voltage for untreated and H_2O_2/NH_4OH solution treated NW sample-based solar cells.

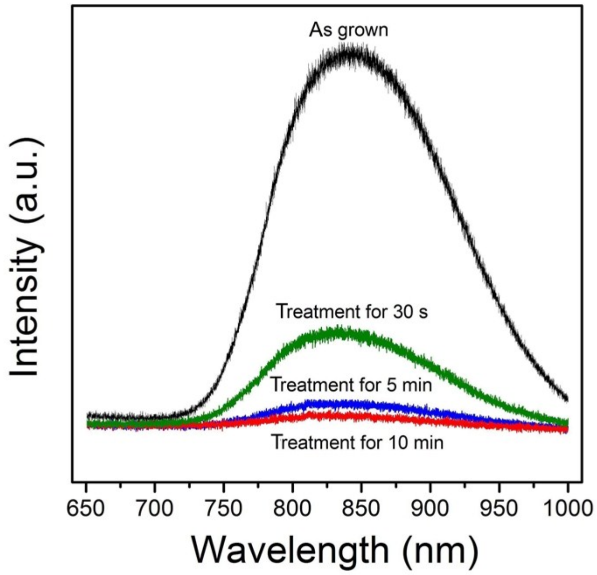

Fig. S3: PL spectra of the as-grown TiO_2 NW arrays after being treated with H_2O_2/NH_4OH solution for different time and annealed at 723 K in oxygen environment for 30 min.

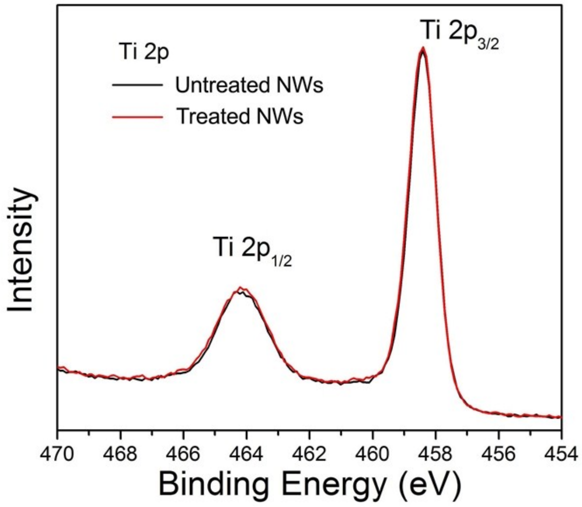

Fig. S4: XPS patterns of TiO_2 NW arrays with and without treatments.

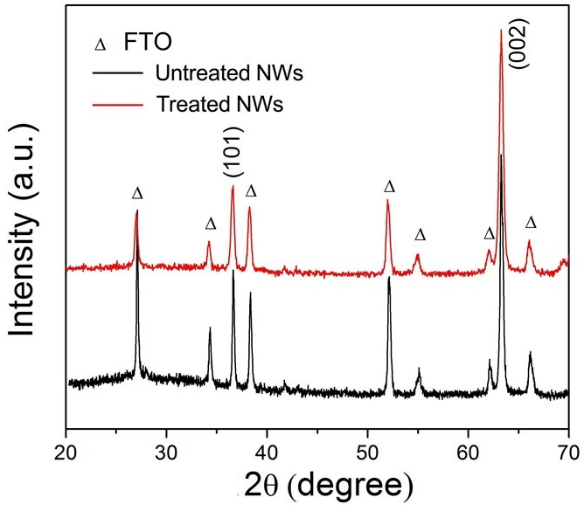

Fig. S5: XRD patterns of TiO_2 NW arrays with and without treatments.

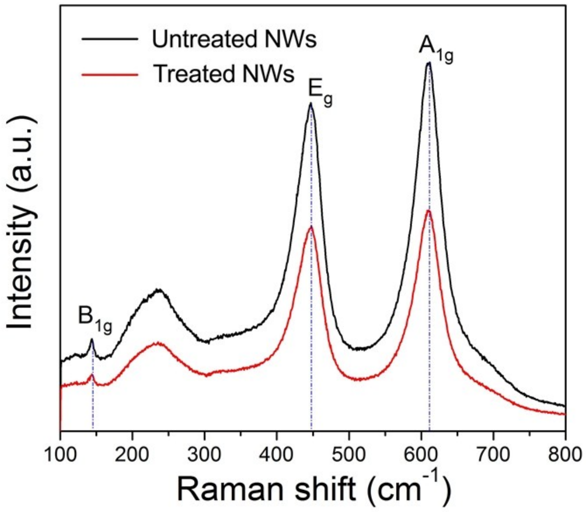

Fig. S6:. Raman spectra of TiO_2 NW arrays with and without treatments.

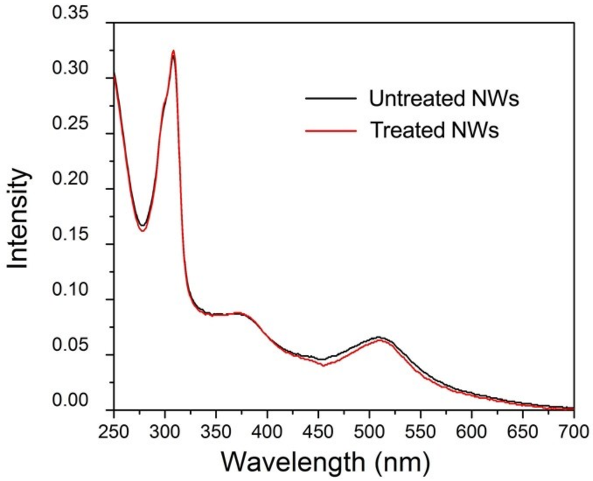

Fig. S7: UV-VIS spectra of dye solutions that desorbed from TiO_2 NW arrays with and without treatments.
